# Editorial: Schools as an arena for health-promoting physical activity

**DOI:** 10.3389/fspor.2025.1697382

**Published:** 2025-09-15

**Authors:** Svein Barene, Domenico Martone, Ole Petter Hjelle, Malte Nejst Larsen

**Affiliations:** 1 Department of Public Health and Sport Sciences, University of Inland Norway, Elverum, Norway; 2 Department of Economics, Law, Cybersecurity and Sports Sciences, University of Naples Parthenope Naples, Naples, Italy; 3 Department of Health and Nursing Sciences, University of Inland Norway, Elverum, Norway; 4 Department of Sports Science and Clinical Biomechanics, Faculty of Health Sciences, University of Southern Denmark, Odense, Denmark

**Keywords:** physical activity, school, health promotion, interventions, mental health, implementation strategies, school environment

Regular physical activity among children and adolescents is linked to better physical fitness (1), cardiometabolic health (2), bone health (3), academic performance (4), executive function (5), quality of life (6), mental health and overall well-being (7). Yet, sedentary lifestyles are on the rise globally, driven by factors such as increased screen time and motorized transport (8).

Given this trend, schools—attended by nearly all children in most countries, represent a powerful and accessible setting for promoting health-enhancing physical activity. However, their effectiveness depends not only on programme availability but also on key delivery elements that determine quality and impact (9).

This Research Topic brings together 23 contributions examining how physical education (PE) and school-based opportunities for physical activity can be structured, implemented, and evaluated within a health-promotion framework. In the following sections, these contributions are grouped into four interrelated themes, each illustrating different approaches to making schools supportive, engaging, and sustainable environments for regular physical activity.

## Quality and implementation of PE and school-based physical activity opportunities

Several studies underscore that successful programmes depend on thoughtful design and execution. In Denmark, Hartman et al. adapted the Resistance Training for Teens (RT4T) program in a small-scale study and found increased self-efficacy among lower secondary school students, though sustaining motivation was challenging. Similarly, Koch et al. evaluated the FIT FIRST 10 multi-sport programme in a cluster RCT and found high fidelity in both full and reduced intervention formats, with implementation constrained by time, facilities, and modest teacher motivation.

Using an ecological perspective, Hoy et al. observed in Swedish middle schools that opportunities for physical activity were often negotiated within school cultures in ways that tended to favour already active pupils. In a longitudinal evaluation, Vedøy et al., reported from a three-year longitudinal evaluation of the FYSAK model in Norway that one model school showed higher device-measured activity and greater adherence to national recommendations than comparison schools, particularly on weekdays.

These findings highlight that effective programme delivery relies on contextual relevance, seamless integration into daily school life, and sustained commitment from teachers.

## Whole-school and multicomponent approaches

School culture and environment can shape how children engage in physical activity. Lemberg et al. found that daily outdoor recess, when embedded in a supportive school setting, was linked to more positive attitudes toward physical activity among pupils and parents, as well as greater participation in leisure-time activity. In a Swiss context, Ferrari et al. evaluated an “open gym” lunchtime programme in all-day primary schools, reporting 37% participation across a socio-culturally diverse pupil group, with higher attendance among boys but increased engagement among girls.

Pupil participation in shaping activities emerged as a theme in Pardali et al., who reported that opportunities to influence physically active learning and recess varied with age and school context. The influence of family was evident in Vorlíček et al., where device-based and survey data showed that parental activity habits and active transport were associated with children’s school-day activity levels.

Taken together, these studies suggest that whole-school strategies which combine supportive environments, meaningful pupil involvement, and family engagement may contribute to sustaining physically active school cultures.

## Inclusive and supportive strategies for participation and wellbeing

Creating school environments that are both inclusive and supportive can help ensure that all pupils, regardless of ability, background, or personal circumstances, have opportunities to engage in meaningful physical activity and experience associated wellbeing benefits. Within this broader focus, several studies examined how school-based physical activity can be promoted among pupils with different needs and situations. Bertills and Björk described how PE teachers facilitate participation for students with disabilities through inclusive mindsets, careful preparation, and adaptations, supported by ongoing teacher–student communication. Wiklund et al. found that school nurses, who are well placed to promote children’s physical activity, used varied approaches to motivate participation but lacked common guidelines, which may contribute to unequal access to opportunities for activity. Altenburg et al. added the perspective of pupils themselves, identifying outcomes they considered most important in school-based health programmes, including “being healthy,” “having fun,” and “feeling happy.”

Jochum et al. evaluated a six-week adapted folk-dance programme for at-risk adolescents, reporting improvements in mental well-being and reduced sedentary time, supported by qualitative findings indicating high enjoyment and increased willingness to be active. Furthermore, Grasaas et al.  explored the relationship between physical activity levels and satisfaction with life, finding that self-efficacy partially mediated this relationship. Gender differences suggested the need for tailored approaches. In line with these findings, Olsen et al. reported that the FIT FIRST FOR ALL intervention in the Faroe Islands improved health-related quality of life, particularly among boys and younger pupils.

Together, these studies highlight that inclusive and supportive strategies can promote both participation and wellbeing, especially when interventions are responsive to pupils’ diverse needs and circumstances.

## Evaluation, evidence, and policy implications

Evaluation studies have provided important insights into the design, delivery, and sustainability of school-based physical activity. Barene et al. described the MOVE12 pilot, using the Intervention Mapping protocol to integrate short daily activity breaks into Norwegian upper secondary lessons, emphasizing motivation, environmental support, and stakeholder involvement. Bloch et al. found that an additional weekly PE hour produced sustained improvements in movement, fitness, and motor competence one year later.

Evidence syntheses further strengthen the knowledge base. Li et al. meta-analysed links between fine motor skills and academic performance, especially mathematics. Liersch et al. reviewed studies showing cognitive benefits from embedding physical activity across school contexts. Becerra-Patiño et al. mapped research on the 20 m shuttle run, underscoring its role as a standardised fitness measure.

Policy contexts influence implementation. Reyes Rodríguez and Martínez Rojas examined Chile’s decision to make PE optional in upper secondary school, potentially conflicting with public health aims. Toscani and Pedersen found Italian teachers’ fitness estimates often misaligned with measured results, suggesting a need for better assessment tools. Andermo et al. identified fun, inclusivity, timetable integration, and leadership as key to extending school-day activity. Nielsen et al. highlighted organisational factors, such as leadership support and resource coordination, that shape early implementation.

Collectively, these studies demonstrate that effective scaling of school-based physical activity depends on rigorous evaluation, a strong evidence base, and supportive policy frameworks.

## Concluding remarks

The 23 contributions illustrate the potential of schools as arenas for health-enhancing physical activity, with evidence of sustained increases in activity (Vedøy et al.), improvements in well-being (Jochum et al.), gains in self-efficacy (Hartman et al.), and varied, context-dependent outcomes across age, gender, and ability. The diversity of methods, from RCTs and ethnographies to meta-analyses, strengthens the evidence base, yet variability in effects, implementation challenges (Koch et al.), and persistent equity gaps (e.g., Grasaas et al., Bertills and Björk) highlight the importance of contextual adaptation and inclusive delivery.

Key challenges include limited time and resources, misaligned policies (Reyes Rodríguez and Martínez Rojas), and disparities in access and participation (Ferrari et al., Hoy et al.). Moving forward, promising directions include strengthening school–family links (Vorlíček et al.), embedding pupil perspectives in programme design (Pardali et al.), and capturing both cognitive and physical outcomes (Li et al., Liersch et al.). Expanding research in older adolescents and underrepresented settings, combined with rigorous and scalable evaluation designs, will be key to translating potential into sustained practice.
