# Correction: Editorial: Schools as an arena for health-promoting physical activity

**DOI:** 10.3389/fspor.2026.1867406

**Published:** 2026-05-15

**Authors:** Svein Barene, Domenico Martone, Ole Petter Hjelle, Malte Nejst Larsen

**Affiliations:** 1 Department of Public Health and Sport Sciences, University of Inland Norway, Elverum, Norway; 2 Department of Economics, Law, Cybersecurity and Sports Sciences, University of Naples Parthenope Naples, Naples, Italy; 3 Department of Health and Nursing Sciences, University of Inland Norway, Elverum, Norway; 4 Department of Sports Science and Clinical Biomechanics, Faculty of Health Sciences, University of Southern Denmark, Odense, Denmark

**Keywords:** physical activity, school, health promotion, interventions, mental health, implementation strategies, school environment

## Introduction

Reference 1 was erroneously written as Zhou X, Li J, Jiang X. Effects of different types of exercise intensity on improving health-related physical fitness in children and adolescents: a systematic review. *Sci Rep*. (2024) 14(1):14301. 10.1038/s41598-024-62449-3. It should be Zhou X, Li J, Jiang X. Effects of different types of exercise intensity on improving health-related physical fitness in children and adolescents: a systematic review. *Sci Rep*. (2024) 14(1):14301. 10.1038/s41598-024-64830-x.

Reference 2 was erroneously written as Men J, Zhu G, Li Y, Wu S, Yu Z, Wang P, et al. Impact of exercise on cardiovascular disease risk in overweight or obese children and adolescents: a systematic review and meta-analysis. *BMC Sports Sci Med Rehabil*. (2025) 17(1):225. 10.1186/s13102-025-01013-y. It should be Men J, Zhu G, Li Y, Wu S, Yu Z, Wang P, et al. Impact of exercise on cardiovascular disease risk in overweight or obese children and adolescents: a systematic review and meta-analysis. *BMC Sports Sci Med Rehabil*. (2025) 17(1):225. 10.1186/s13102-025-01228-w.

Reference 4 was erroneously written as Visier-Alfonso ME, Sánchez-López M, Álvarez-Bueno C, Ruiz-Hermosa A, Nieto-López M, Martínez-Vizcaíno V. Mediators between physical activity and academic achievement: a systematic review. *Scand J Med Sci Sports*. (2022) 32(3):452–64. 10.1111/sms.14114. It should be Visier-Alfonso ME, Sánchez-López M, Álvarez-Bueno C, Ruiz-Hermosa A, Nieto-López M, Martínez-Vizcaíno V. Mediators between physical activity and academic achievement: a systematic review. *Scand J Med Sci Sports*. (2022) 32:452–64. 10.1111/sms.14107.

Reference 6 was erroneously written as Bermejo-Cantarero A, Sanchez-Lopez M, Alvarez-Bueno C, Redondo-Tebar A, Garcia-Hermoso A, Martinez-Vizcaino V. Are physical activity interventions effective in improving health-related quality of life in children and adolescents? A systematic review and meta-analysis. *Sports Health*. (2024) 16(6):877–85. 10.1177/19417381241232621. It should be Bermejo-Cantarero A, Sanchez-Lopez M, Alvarez-Bueno C, Redondo-Tebar A, Garcia-Hermoso A, Martinez-Vizcaino V. Are physical activity interventions effective in improving health-related quality of life in children and adolescents? A systematic review and meta-analysis. *Sports Health*. (2024) 16(6):877–85. 10.1177/19417381231190885.

Reference 7 was erroneously written as Hale GE, Colquhoun L, Lancastle D., Lewis N, Tyson PJ. Physical activity interventions for the mental health and well-being of adolescents—a systematic review. *Child Adolesc Ment Health*. (2021) 26(4):357-68. 10.1111/camh.12448. It should be Hale GE, Colquhoun L, Lancastle D, Lewis N, Tyson PJ. Review: Physical activity interventions for the mental health and well-being of adolescents—a systematic review. *Child Adolesc Ment Health*. (2021) 26(4):357-68. 10.1111/camh.12485.

The original version of this article has been updated.

